# Exploring Mental Help-Seeking Behaviors, Health, and Well-Being in Rural Veterans with Chronic Health Conditions: A National Rural Health Study

**DOI:** 10.3390/healthcare13233108

**Published:** 2025-11-28

**Authors:** Jimena Ortiz, Beatrice Lee, Jeannie Concha, John Barnas, Jim Yates, Emre Umucu

**Affiliations:** 1 Department of Public Health Sciences, The University of Texas, El Paso, TX 79968, USA; 2 Rehabilitation Sciences, The University of Texas, El Paso, TX 79968, USA; 3 Michigan Center for Rural Health, East Lansing, MI 48823, USA

**Keywords:** rural veterans, well-being, help-seeking

## Abstract

**Highlights:**

**What are the main findings?**

- Willingness to seek psychological help was positively associated with the well-being of rural Veterans.
- Many rural Veterans experience high levels of mental health conditions.

**What is the implication of the main finding?**

- Policymakers should invest in rural settings to improve mental health service delivery for veterans living in rural settings.
- More research should investigate how to reduce reluctance to seek psychological help among rural veterans.

**Abstract:**

**Background/Objective:** Veterans in the United States have increased significantly, with a substantial proportion residing in rural areas. This study aims to examine the relationship between help-seeking behaviors and well-being, controlling demographic variables and service-connected disability status. It also explores mental health–seeking behaviors, health outcomes, and overall well-being among rural Veterans with existing health conditions. **Methods:** Data was collected through surveys distributed across multiple channels, including social media platforms. **Results:** Results indicated that willingness to seek psychological help was positively associated with the well-being of rural Veterans. **Conclusions:** These findings underscore the critical importance of both willingness to seek mental health support and access to mental health and substance use care services for rural Veterans.

## 1. Introduction

Roughly 4.4 million Veterans live in rural America, and of these, about 2.7 million are enrolled in the VA healthcare system [1,2]. Many veterans prefer rural life for practical and personal reasons (e.g., lower housing costs, proximity to family). Residing in rural areas also comes with certain challenges, affecting health and well-being of this population. Although about 58% of rural veterans experience at least one service-connected disability, most are reluctant to receive services or have no access to services [1,3]. Given that Veterans face an increased risk of biopsychosocial challenges, including social isolation, homelessness, sleep disturbances, substance use problems, and mental health conditions [4,5,6,7,8,9,10,11,12,13,14], it is important to promote access to healthcare services and encourage willingness to seek care, particularly among rural Veterans.

In addition to high rates of service-connected disability among Veteran population regardless where they live [6], they also experience broader socioecological disparities in rural areas, including higher rates of poverty. Rural Veterans are significantly less likely to pursue specialized services such as residential treatment or case management compared to those living in urban areas [3]. In addition, many rural veterans face systemic barriers to care [15,16]. This issue is compounded by widespread hospital closures and healthcare provider shortages in rural regions. In addition, prolonged wait times, often exceeding 40 days for both primary and specialty appointments, further discourage engagement with available services.

Cultural and psychological barriers also play a major role in shaping help-seeking behaviors. Persistent stigma surrounding mental health and substance use contributes to lower treatment utilization and higher risks of depression, substance misuse, and suicide [17]. This stigma operates on multiple levels: public stigma, which reflects societal judgment, and self-stigma, which involves internalized negative beliefs [18]. Military culture has often discouraged open discussion of psychological distress [1,2,3].

Given these challenges, it is critical to examine whether willingness to seek behavioral help is associated with health and well-being outcomes among rural Veterans with chronic conditions. This study, therefore, aims to examine the relationship between help-seeking behaviors and well-being, controlling demographic variables and service-connected disability status. Insights from this research can inform targeted interventions and guide policies aimed at improving healthcare access, mental health outcomes, and quality of life for rural Veterans. Our research question is as follows: Is willingness to seek behavioral help positively associated with health and well-being outcomes among rural Veterans with chronic conditions, after controlling demographic factors and service-connected disability status? We hypothesized that rural Veterans with chronic conditions who demonstrate a greater willingness to seek behavioral help will report higher levels of health and well-being, even when demographic variables and service-connected disability status are controlled for.

## 2. Materials and Methods

This project is part of the **I**mproving **R**ural **E**nrollment, **AC**cess, and **H**ealthcare in Rural Veterans (I-REACH Rural Veterans) Program developed by the PI (EU) and funded by the Federal Office of Rural Health Policy Rural Veterans Health Access Program of the U.S. Department of Health and Human Services. Approval from the Institutional Review Board’s ethics committee was obtained prior to the study. Participants provided informed consent before participating in the survey, ensuring they were fully aware of the study’s purpose and their rights as participants. Participants were eligible for our study if they met all of the following criteria: (a) a Veteran who is 18 years old or older, and (b) living in a rural area in the U.S. The survey was distributed through multiple channels, including social media. Specifically, the project coordinator (JY) shared the survey with organizations serving rural Veterans. Additionally, we shared the survey link with community leaders via social media and email. We also collaborated with other funded projects serving rural Veterans nationwide to disseminate the survey. Data quality was checked utilizing attention check items (e.g., “Select correct responses: five plus 2 = seven.”; “Select the color option below: Car.”). For the purpose of this study, we only extracted veterans who had any self-reported physical or mental health condition, resulting in a total of 400 veterans with physical and mental health conditions living in rural settings.

Participants’ mean age was 36.05 (SD = 11.43). The majority of participants were male (*n* = 316; 79%). Most participants were non-Hispanic White (*n* = 275; 68.8%), followed by Black (*n* = 70; 17.5%), American Indian or Alaska Native (*n* = 25; 6.3%), Native Hawaiian or Pacific Islander (*n* = 14; 3.5%), Asian (*n* = 10; 2.5%), and others (*n* = 6; 1.6%). A total of 84 participants identified as Hispanic (21%), and most participants had at least high school degree (96.3%). Most participants were employed (*n* = 284; 71%). The majority of participants were married (60.5%), followed by single (28.5%), while smaller proportions were divorced (3.5%), widowed (2.0%), separated (1.8%), or cohabitating (3.5%).

A demographic questionnaire collected information on participants’ age, gender, race, and education. Employment status was assessed with a single item (e.g., “What is your current employment status?”), and responses were coded dichotomously as employed or unemployed. Clinical status was also assessed using a single-item question (e.g., “Do you have any of the following conditions? Please check all that apply”). Participants’ mental health conditions were assessed using a checklist format, where they indicated whether they had any of the listed conditions. Substance use was measured similarly, with participants asked to report whether they had ever used specific substances by marking items on a checklist. The short version of the PERMA-Profiler [19] (Butler & Kern, 2016) was used to assess positive emotion, engagement, relationships, meaning, and accomplishment (PERMA) [20]. Each item was rated on an 11-point scale ranging from 0 (never/not at all) to 10 (always/completely). The short version of the PERMA-Profiler demonstrates excellent internal consistency, with a Cronbach’s alpha of 0.90. Participants’ mental health help-seeking behavior was measured using a single item (“I am willing to see a therapist for psychological help.”), rated from 1 (strongly disagree) to 5 (strongly agree) [20].

Descriptive statistics were used to summarize participants’ demographic and health characteristics. To address the research question, a hierarchical regression analysis was conducted to examine the relationship between help-seeking behaviors and well-being, controlling demographic variables and service-connected disability status. In Step 1, demographic covariates were entered (age, gender [1 = female], minority status [1 = minority], and education [1 = at least high school graduate]). In Step 2, the number of mental health conditions and the number of substances use disorders were added. In Step 3, help-seeking behaviors were entered. All analyses were conducted using SPSS version 28.0.

## 3. Results

### 3.1. Descriptive Statistics

Approximately 46% of participants indicated having a service-connected disability. For mental health conditions, reported rates included depression (45%), anxiety (51%), PTSD (26.3%), bipolar disorder (19.3%), substance use disorders (8%), personality disorder (7%), and schizophrenia (5%). Physical health conditions reported were migraines (25.5%), tinnitus (20.3%), paralysis (18.3%), hearing loss (17.5%), musculoskeletal disease (14%), Alzheimer’s disease (2.5%), and other conditions (4.8%). In addition, 26.3% of the sample identified as being homeless or at risk of homelessness. Willingness to seek psychological help was assessed on a 5-point scale, with an average score of 3.81 (SD = 0.96). The mean PERMA well-being score was 6.15 (SD = 1.83). On average, participants reported 1.61 mental health conditions (SD = 1.20) and 2.37 substance use conditions (SD = 2.05) (Please see Table 1).

### 3.2. Association Between Psychological Help-Seeking and Well-Being

A hierarchical multiple regression was used to identify associates of well-being among rural Veterans. In Model 1, demographic variables (age, gender, minority status, and education level) were entered and accounted for 7.3% of the variance in well-being (R^2^ = 0.073, *p* < 0.001). Age (B = 0.125, *p* = 0.002), female gender (B = 2.67, *p* = 0.016), and minority status (B = 3.68, *p* < 0.001) were significant positive associates of well-being, suggesting that older, female, and minority Veterans reported higher well-being scores.

Model 2 introduced the number of mental health and substance use conditions, significantly improving the model (ΔR^2^ = 0.118, *p* < 0.001), bringing the total explained variance to 19.1% (R^2^ = 0.191, *p* < 0.001). Both mental health conditions (B = −2.07, *p* < 0.001) and substance use conditions (B = −0.65, *p* = 0.002) were significant negative associates of well-being, confirming that increased health conditions were associated with lower well-being.

In Model 3, willingness to seek psychological help was added, further improving model fit (ΔR^2^ = 0.034, *p* < 0.001), bringing the total explained variance to 22.5% (R^2^ = 0.225, *p* < 0.001). Willingness to seek psychological help was a significant positive associates of well-being (B = 1.80, *p* < 0.001), indicating that those who were more willing to seek psychological help reported higher well-being scores.

## 4. Discussion

The purpose of this study is to examine whether willingness to seek psychological help is associated with well-being among rural Veterans, after controlling for sociodemographic and clinical characteristics. Our results revealed that older, female, and minority Veterans reported higher well-being scores, which are somehow inconsistent with previous findings. One study reported that younger, female, and Hispanic Veterans reported the lowest well-being scores [21]. Rural locations often have stronger community ties, social cohesion, and reliance on informal support systems, which can serve as protective factors for well-being [1,2,3,14]. This may partially explain why older, minority, and female Veterans in rural areas reported higher well-being.

The main results of this study revealed that willingness to seek psychological help significantly and positively associated with well-being, indicating that rural Veterans who were more willing to seek psychological help reported higher well-being scores. However, rural Veterans often face challenges with trust in their providers, which can discourage help-seeking and, in turn, reduce well-being. Stigma related to mental illness and substance use remains a major obstacle, frequently exacerbating existing clinical conditions [22].

Exploring rural Veterans’ willingness to seek mental health care in relation to their overall well-being is crucial. Understanding these dynamics can inform policies and targeted interventions to reduce barriers, improve healthcare access, and promote better health outcomes among rural Veterans. Furthermore, because rural Veterans encounter both practical obstacles and stigma when seeking mental health care, it is essential to foster a culture where accessing support is easy and socially accepted. For example, peer-support initiatives may be effective in promoting help-seeking among Veterans, since most Veterans feel more comfortable sharing mental health concerns with peers who have similar military backgrounds.

Several limitations of this study should be acknowledged. First, distributing the survey through media outlets may have introduced selection bias, as individuals who are more comfortable using technology or engaging with online platforms might be overrepresented. Second, the study relied on self-reported data, which can be influenced by recall bias or social desirability bias. In addition, the definition of “living in a rural area in the United States” was not explicitly provided, potentially leading respondents to interpret the term differently based on their own experiences. The sample may also disproportionately include individuals who are already proactive in seeking mental health care, which could limit the generalizability of the findings. Next, because this study used a cross-sectional, correlational design, the results cannot be used to infer causal relationships between variables. Finally, some of our constructs were measured with a single-item measure, a comprehensive scale would better capture the constructs like help-seeking behaviors.

## 5. Conclusions

Our study examined the association between the willingness to seek psychological help and well-being among rural Veterans. Findings revealed that it is critical to increase the willingness to seek mental health support among rural Veterans, which may eventually improve their health and well-being outcomes. Rural Veterans often face challenges with seeking mental health services due to stigma or access to no services; therefore, it is important to increase the outreach for rural Veterans.

## Figures and Tables

**Table 1:** Participant Characteristics, Health Conditions, and Well-being Measures.

Variable	Value
**Demographics**	
Age (years), M (SD)	36.05 (11.43)
Gender (n, %)	
Male	316 (79.0)
Female	82 (20.5)
Race (n, %)	
White	275 (68.8)
Black/African American	70 (17.5)
American Indian/Alaska Native	25 (6.3)
Native Hawaiian/Pacific Islander	14 (3.5)
Asian	10 (2.5)
Hispanic Origin (n, %)	84 (21.0)
Service-connected disability (n, %)	209 (52.3)
**Mental Health Conditions (n, %)**	
Depression	180 (45.0)
Anxiety	204 (51.0)
PTSD	105 (26.3)
Bipolar disorder	77 (19.3)
Personality disorder	28 (7.0)
Schizophrenia	20 (5.0)
Substance use disorder	32 (8.0)
**Physical Health Conditions (n, %)**	
Migraines	102 (25.5)
Tinnitus	81 (20.3)
Paralysis	73 (18.3)
Hearing loss	70 (17.5)
Musculoskeletal disease	56 (14.0)
Alzheimer’s disease	10 (2.5)
Other conditions	19 (4.8)
Chronic condition (any)	392 (98.0)
**Other Measures**	
Willingness to seek help, M(SD)	3.81 (0.96)
PERMA, M(SD)	6.15 (1.83)

## Data Availability

The dataset used and analyzed in this study is available from the corresponding author. The data are not publicly available due to ethical restrictions.
